# The *Tomato spotted wilt virus* (TSWV) Genome is Differentially Targeted in TSWV-Infected Tomato (*Solanum lycopersicum*) with or without *Sw-5* Gene

**DOI:** 10.3390/v12040363

**Published:** 2020-03-26

**Authors:** Cristian Olaya, Stephen J. Fletcher, Ying Zhai, Jonathan Peters, Paolo Margaria, Stephan Winter, Neena Mitter, Hanu R. Pappu

**Affiliations:** 1 Department of Plant Pathology, Washington State University, Pullman, WA 99164, USA; cristian.olayaarias@wsu.edu (C.O.); ying.zhai@wsu.edu (Y.Z.); 2 Centre for Horticultural Science, Queensland Alliance for Agriculture and Food Initiative, St. Lucia, QLD 4069, Australia; s.fletcher@uq.edu.au (S.J.F.); jonathan.peters@uq.edu.au (J.P.); n.mitter@uq.edu.au (N.M.); 3 Deutsche Sammlung von Mikroorganismen und Zellkulturen (DSMZ, German Collection of Microorganisms and Cell Cultures GmbH), 38124 Braunschweig, Germany; Paolo.Margaria@dsmz.de (P.M.); Stephan.Winter@dsmz.de (S.W.)

**Keywords:** tospoviruses, *Tomato spotted wilt tospovirus* (TSWV), viral-small-interfering RNAs (vsiRNAs), *Sw*-5(+) and *Sw*-5(−), tomato, deep sequencing

## Abstract

Tospoviruses cause significant losses to a wide range of agronomic and horticultural crops worldwide. The type member, *Tomato spotted wilt tospovirus* (TSWV), causes systemic infection in susceptible tomato cultivars, whereas its infection is localized in cultivars carrying the *Sw-*5 resistance gene. The response to TSWV infection in tomato cultivars with or without *Sw*-5 was determined at the virus small RNA level in the locally infected leaf. Predicted reads were aligned to TSWV reference sequences. The TSWV genome was found to be differentially processed among each of the three-viral genomic RNAs—Large (L), Medium (M) and Small (S)—in the *Sw*-5(+) compared to *Sw*-5(−) genotypes. In the *Sw*-5(+) cultivar, the L RNA had the highest number of viral small-interfering RNAs (vsiRNAs), whereas in the *Sw*-5(−) cultivar the number was higher in the S RNA. Among the three-viral genomic RNAs, the distribution of hotspots showed a higher number of reads per million reads of vsiRNAs of 21 and 22 nt class at the 5′ and 3′ ends of the L and the S RNAs, with less coverage in the M RNA. In the *Sw*-5(−) cultivar, the nature of the 5′ nucleotide-end in the siRNAs varied significantly; reads with 5′-adenine-end were most abundant in the mock control, whereas cytosine and uracil were more abundant in the infected plants. No such differences were seen in case of the resistant genotype. Findings provided insights into the response of tomato cultivars to TSWV infection.

## 1. Introduction

Tospoviruses belong to the genus *Orthotospovirus*, family *Tospoviridae* (order *Bunyavirales*), the sole genus of one of the three plant-infecting families in the order [1,2,3,4]. *Tomato spotted wilt virus* (TSWV), the type species, is transmitted by thrips, which have contributed to the worldwide dispersion and became one of the most important viral-vector complexes for agriculture and food security [5,6,7]. The tospovirus genome consists of three segmented negative/ambisense RNA molecules named according to their size: L (large), M (medium), and S (small) [8,9]. The L segment, with a size of 8.9 kb, encodes an RNA-dependent RNA polymerase (RdRp) in the viral-complementary sense orientation; the M RNA with a size of 4.5 kb, encodes the precursors of the glycoproteins G_N_ and G_C_ in the virion complementary sense and the movement protein, NSm, in the virion sense orientation; and the S with a size of 2.9 kb encodes the nucleocapsid protein (N) in the virion complementary sense, and the silencing suppressor protein (NSs) in the virion sense orientation [6,8,9].

One of the most effective tactics to reduce the impact of viral diseases include growing virus resistant cultivars. The success of this approach relies on the availability of resistant genes in cultivated or wild relatives of a crop. The most common mechanism of natural plant resistance to virus infection is the hypersensitive response (HR) [10,11]. HR leads to rapid death of cells surrounding the viral infection thereby limiting the viral cell to cell spread and the subsequent spread of the virus through rest of the plant. The HR manifests itself as local lesions at the site of virus entry. This response is triggered by specific recognition of the virus based on matching gene products of plant and virus. In case of tospoviruses and specifically TSWV, the principal sources of HR-based resistance are the dominant genes *Sw-5* and *Tsw*, in tomato and pepper, respectively [12]. For *Sw*-5, several homologs were found in the tomato genome, but *Sw*-5b, provides broad and durable resistance and has been extensively studied due to this functionality [13]. The TSWV product that triggers the resistance response (*Avr* determinant) of *Sw-5b*-mediated resistance is the NSm, encoded in the M segment of TSWV [13,14,15]. This was demonstrated by inoculating *Sw*-5 tomato plants with assorted viruses containing the L and S segments from a TSWV resistance-inducing (RI) isolate, and the M segment from a TSWV resistant breaking (RB) isolate [16].

In the last decade, another important mechanism has been uncovered and its understanding is giving valuable information to generate new strategies to resist viral infections. This mechanism is based on post-transcriptional gene silencing or RNA-silencing found in plants, fungi, and animals. RNA-silencing (also known as RNA interference, RNAi) is a conserved defense mechanism that suppresses the expression of nucleic acids from viruses, transposons, or host genes that need to be regulated [17,18].

During the RNAi phenomenon, double-stranded RNA (dsRNA, such as the viral replicative form) or hairpin RNA (hpRNA) are cleaved by Dicer-like proteins (DCL) into small interfering RNAs (siRNA) or microRNA (miRNA) of 21–24 nucleotides [19,20]. From the resulting small dsRNA molecules, one strand is loaded into the argonaute, thereby inducing the assembly of the RNA-induced silencing complex (RISC), while the other strand is degraded. The argonaute-loaded siRNA is then used as a guide to cleave the target analogous strands [21,22,23,24].

The virus-derived small RNAs (vsiRNAs) are associated with antiviral immunity via silencing the viral genomic RNA [25]. Studies of vsiRNAs profiling have shown some characteristics such as vsiRNAs being derived mainly in a polarity manner, vsiRNAs concentrated in a limited number of hotspots, and presence of mismatches upon their affinity with the RNA-silencing suppressor protein [25]. Moreover, the tospoviral RNA-silencing suppressor binds to short and long RNAs, and the tospovirus genome is processed by the RNA-silencing machinery of its plant hosts [26,27,28,29]. Several studies on the TSWV siRNA profiling have been published on different hosts such as tomato, peanut, *N. benthamiana*, but also in its insect vector [30,31,32,33,34]. Mitter et al. [29], in systemically TSWV-infected, non-inoculated leaves of *N. benthamiana* and susceptible tomato (cv. Sunny), found the vsiRNA profiles similar in both hosts in terms of relative abundance of 21, 22, and 24 nt class size. However, the number of vsiRNA reads detected was higher in tomato than in *N. benthamiana*. The mapping of viral siRNAs to the TSWV genome suggested that the hotspots were distributed depending on the virus isolate and the activity of the viral silencing suppressor, NSs [28,29]. The higher amount of vsiRNAs corresponded to the M and S RNAs, and a fewer representation of the intergenic regions and the L RNA [26,27,28,29]. This can be explained because L RNA is less frequent in infected cells than the M and the S RNA [27,28]. Additionally, Mitter et al. [29] found more abundance of vsiRNAs matching the G_N_/G_C_ gene than the NSm in the M RNA, contrasting more processing of the NSs gene than the N in the S RNA. Another interesting conclusion of this study was that the vsiRNAs processing for TSWV genes was higher in the viral sense than in the viral-complementary sense, but the opposite for the NSs.

A better understanding of the different molecular mechanisms during the interaction between the virus and the host silencing machinery is useful for the development of novel technologies to control the virus. The vsiRNA outputs of TSWV in susceptible tomato and in systemically infected leaves have been studied previously. However, it is unknown how the RNAi machinery operates against TSWV in resistant genotypes and in a local infection scenario. Moreover, the output of RNAi in resistant cultivars under incompatible infections has not been addressed. the objective of this study was to determine the small RNA profiles of TSWV in infected tomato cultivars resistant [*Sw*-5(+)] and susceptible [*Sw*-5(−)] in the locally infected leaves.

## 2. Methods

### 2.1. Virus and Plant Inoculations

TSWV was originally isolated from a naturally infected peanut (*Arachis hypogea*) plant which was mechanically transferred to and maintained on *Datura stramonium*. Infected leaves were kept frozen at −80 °C as a lab stock. TSWV was subsequently maintained on *N. benthamiana* plants. Tomato (*Solanum lycopersicum*) varieties, Red Defender [*Sw*-5(+); Batch: Q45093, year 2008] resistant to TSWV, and Marglobe [*Sw*-5(−); Batch: 15705, year 2007] susceptible to TSWV, were sown in trays in a growth chamber and transplanted to individual pots in a greenhouse. All plants were kept in nylon mesh tents to protect them against insect infestation including thrips. Greenhouse conditions were 26 °C with 16 h day and 8 h night. Twenty-eight days post-emergence, the first three fully expanded leaves of tomato plants were manually inoculated with TSWV inoculum. Frozen TSWV-infected *N. benthamiana* tissue was homogenized by grinding in 0.01 M sodium phosphate buffer (pH 7.0) containing 0.4% β-Mercaptoethanol and carborundum. Leaves #1 to #3 of control tomato plants of both varieties were mock-inoculated with buffer.

The inoculated leaf #3 was collected eight days post-inoculation (dpi, when first local lesions developed), immediately frozen in liquid nitrogen and then stored at −80 °C. The tissues were ground in liquid nitrogen and sets of 100 mgs were stored in 2 ml tubes for DAS-ELISA (Agdia Inc.; Elkhart, IN, USA) and RNA isolation. Treatments consisted of resistant (Red Defender) and susceptible (Marglobe) tomato cultivars that were mock- or TSWV-inoculated, with three biological replicates per each treatment.

### 2.2. DAS-ELISA

TSWV levels were tested by DAS-ELISA using a commercial kit (Agdia Inc.; Elkhart, IN, USA) following manufacturer’s instructions. Healthy and TSWV-infected *N. benthamiana* were used as negative and positive controls, respectively.

### 2.3. RNA Isolation

Based on the ELISA test, nine plants of each treatment with absorbance values close to the average were selected and mixed into groups of three to get three biological replicates. Thus, each biological replicate consisted of a mixture of total RNA of three samples. Total RNA was extracted using Trizol (Invitrogen, USA) following the manufacturer’s instructions and then treated with Turbo-DNase (Invitrogen). Total RNA was quantified using Nanodrop, replicates were mixed and RNA quality numbers (RQN) were measured in a fragment analyzer at the Washington State University (WSU) Genomic Core Facilities.

### 2.4. Deep Sequencing

RNA integrity was determined with a fragment analyzer (Agilent Technologies, Waldbronn, Germany), and samples with more than 5.0 RQN were sent to the Beijing Genomic Institute (BGI) for library construction and small RNA sequencing using Illumina HiSeq 4000, with a single-end library read length of 50 bp.

### 2.5. Bioinformatic Analysis

Raw data were filtered using an in-house BGI method including removal of the low-quality sequences, 5′ adaptor contaminants from the 50 nucleotide tags. Length of small RNAs between 18 and 30 nucleotides were used for the analysis. Quality Control analysis of clean reads was also checked using the platform CLC Genomics Workbench, version 8.0. (Qiagen, USA) for each treatment. Bulk of contigs with all the reads (de novo assembly) were predicted and compared to *Solanum lycopersicum* reference genome (*Solanum lycopersicum* assembly SL2.50) using BLAST. Contigs were mapped back to the reads of each treatment to the assembled contigs.

Collapsed to identical sequences with maintained counts using the FASTX-Toolkit package, only reads with lengths between 18 and 30 nt were retained for alignment normalization. Alignment of collapsed reads to reference sequences was performed using the Small Complementary RNA Mapper (SCRAM) software package which allowed for exact matches to the reference sequence only [35]. Normalization of aligned read count at each position was calculated as reads aligned per million reads between length 18 and 30 nt in the collapsed read file, (robust quantitative comparison between alignments).

TSWV siRNAs were aligned to the reference genome L RNA—NC_002052.1; M RNA—NC_002052.1; S RNA—NC_002052.1 using CLC Genomics Workbench, to assemble the genome of our isolate and use it as a reference to determine the viral siRNA profile and predict the hotspots. To the test the accuracy of the prediction, siRNAs were aligned to L RNA sequence (KP827649.1) of the TSWV isolate used in this study.

### 2.6. Primer Design

For real-time PCR, TSWV gene-specific primers were designed using the parameters suggested by Thornton and Basu [36]. In short, sequences of the gene of interest were designed in PRIMER-BLAST [37], then verified in Beacon Designer 7.0 software (Premier Biosoft International, Palo Alto, CA, USA) for self and cross complementarity and secondary structures. Finally, the amplicons predicted with the primers were tested for loop secondary structures formation at 60 °C and 3 mM magnesium concentration in UNAFold software at IDTdna website (www.idtdna.com/UNAFold). TSWV-RdRp, G_N_/G_C_, NSm, and NSs primers (from this study) amplify the RNA transcript for each gene as well as the corresponding genomic RNAs, for this reason, we refer to the templates as TSWV-RdRp, G_N_/G_C_, NSm, and NSs (Supplementary Table S1). Similar annotation was used for the TSWV-N primers by Rotenberg et al. [38] and Badillo-Vargas et al. [39].

Primers from other reports, TSWV-N primers [38], tomato reference genes Ubiquitin 3 (UBI), Glyceraldehyde 3-phosphate dehydrogenase (GAPDH), Uridylate kinase (UK) [40] and Elongation factor 1-alpha (EF1a) [41], were tested for the experimental conditions (Supplementary Table S1). Primers were first tested by end-point PCR to determine nonspecific amplification or dimer formation. In cases where products were not obtained at the annealing temperature tested, gradient-PCR was used.

### 2.7. RT-qPCR of TSWV Genes

RNA was isolated from tissue stored at −80 °C from the same samples and same combinations that were sent for deep sequencing, following Trizol extraction method followed by treatment with Turbo-DNase.

Complementary DNA was made with the iScript Reverse Transcription Supermix (Bio-Rad, Hercules, CA) following the instructions. Briefly, for a 20 µL volume reaction of 4 µL of 5× iScript were mixed with the volume containing 1 µg of RNA, plus nuclease free water to the final volume. Incubation conditions consisted of 5 min at 25 °C for priming, followed by 30 min at 42 °C for reverse transcription, with a final incubation at 5 min at 85 °C for inactivation. cDNA was used immediately for either end-point PCR, qPCR or stored at −20 °C for further assays. qPCR was performed with the SSoAdvanced Universal SYBR Green Supermix (Bio-Rad) in 20 µL reaction containing 10 µL of 2× SSoAdvanced Universal SYBR Green Supermix (Bio-Rad), 2 µL of 50 ng cDNA, 0.7 µL (10 µM) of each primer and 6.6 µL of nuclease free water. The qPCR reactions were carried out in iCycler (Bio-Rad); the program began with DNA denaturation at 95 °C for 30 sec, followed by 40 cycles of denaturation at 95 °C for 15 sec and annealing and extension at 60 °C for 30 sec. Melting curve 60 to 95 °C at 5 sec 0.5 °C per cycle. Elongation factor 1-alpha (EF1alpha) and Glyceraldehyde 3-phosphate dehydrogenase (GAPDH) were chosen for normalization as reference control [40,41]. The ∆Ct method was used to determine relative gene expression. Statistical analysis of the gene expression was carried out in JMP software (version 8.0, SAS institute, Inc.; Cary, NC, USA).

### 2.8. Data Analysis

Relative expression values were determined by fitting to a normal distribution and assessing the goodness of fit with a Shapiro–Wilks W test. One-way ANOVA was carried out and significant differences were compared with Least Significant Difference (LSD) test. The software used was JMP (version 8.0, SAS institute, Inc.; Cary, NC, USA). siRNA 5′ nucleotide enrichment was compared with pairwise t-tests with Bonferroni *p*-value correction using custom Python scripts.

## 3. Results

### 3.1. Disease Symptoms of Sw-5(−) and Sw-5(+) Genotypes

**Figure 1:**
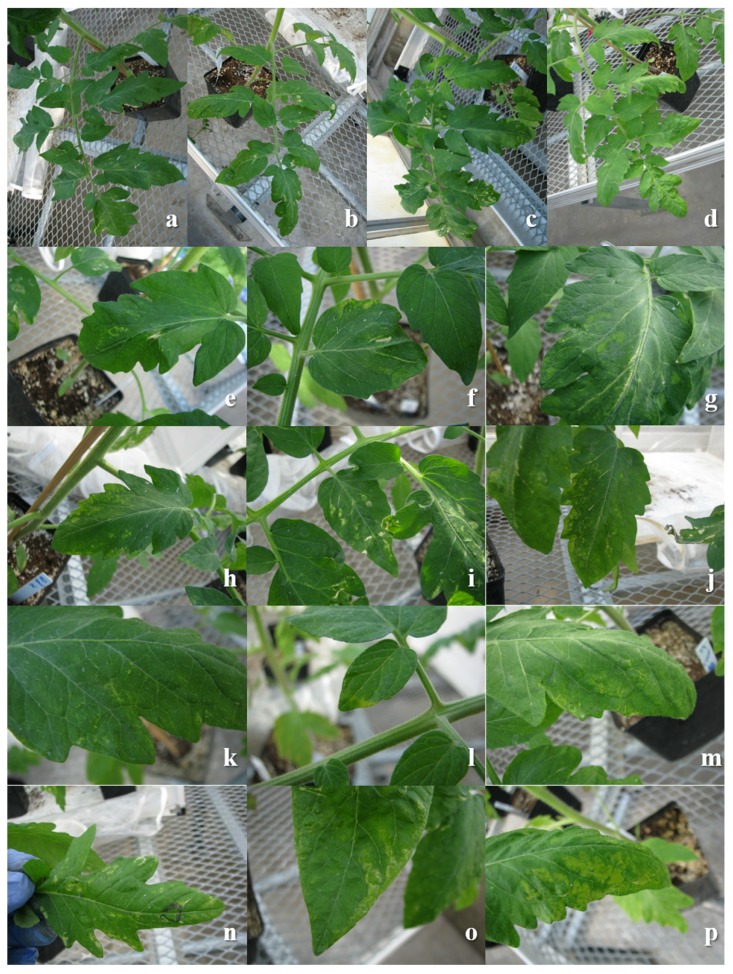
Symptoms expressed on leaf # 3 of tomato cv. Red Defender *Sw*-5(+) or cv. Marglobe *Sw*-5(−) 8 days post-inoculation with *Tomato spotted wilt virus* (TSWV) or mock-inoculated (phosphate buffer). **a**: Red Defender-mock; **b**: Marglobe-mock; **c**: Red Defender-TSWV; **d**: Marglobe-TSWV. **a**–**d**: panoramic view of leaf #3 inoculated with either TSWV or Mock. **e**–**j**: Red Defender expressing TSWV localized symptoms; **k**–**p**: Marglobe expressing TSWV localized symptoms.

TSWV moves systemically in the *Sw*-5(−) genotype, while in the *Sw*-5(+) is restricted to the inoculated leaf (Supplementary Figure S1). Initial symptoms on the susceptible tomato are visible at eight days after inoculation with small chlorotic spots that turn necrotic later, also produce small chlorotic ringspots and mild mosaic (Figure 1). In susceptible tomato, systemic symptoms begin to be visible in younger non-inoculated leaves 14 days after inoculation, which develop into necrotic ringspots, mosaic, necrosis of the petioles and the stem, with plants subsequently becoming stunted. TSWV resistant tomato expresses only local lesions such as chlorotic spots or chlorotic ringspots that turn necrotic (Figure 1). There was no systemic infection or symptom expression as the virus cannot move systemically. The differential effect of TSWV on overall plant growth was quite evident in resistant and susceptible genotypes (Supplementary Figure S1).

### 3.2. Profiles of vsiRNAs

**Figure 2:**
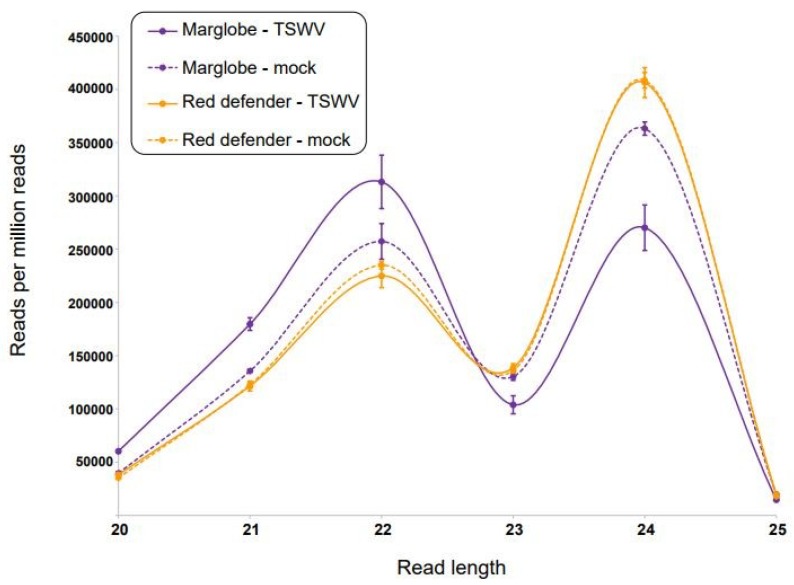
Distribution of the read counts for small RNAs between 20 to 25 nt in length. Each peak corresponds to the average of three biological replicates with standard error bars. Continuous lines are tomato spotted wilt virus (TSWV)-infected treatment, while broken lines are mock control. Purple corresponds to tomato cv. Marglobe, the susceptible genotype *Sw*-5(−), while the yellow line corresponds to cv. Red Defender, the resistant genotype, *Sw*-5(+).

The high-throughput sequencing of the small RNAs on the Illumina HiSeq 4000 yielded 29 to 31 million reads per cDNA library and 650 to 700 million of nucleotides per data set (SRA accession: PRJNA606610). In both tomato genotypes, the mock-inoculated leaves yielded 500,000 more reads than the virus-inoculated leaves. Also, *Sw*-5(+) cultivar gave a slightly higher number of reads than the *Sw*-5(−) (Table 1). The size of the predicted siRNAs ranged from 18 to 30 nt with highest abundance of reads between 20 to 25 nt. The siRNAs were processed differently in the resistant versus susceptible varieties. There is a higher number of siRNAs of 21 and 22 nt in the susceptible compared to the resistant cultivar, but lower when compared 23 and 24 nt. *Sw*-5(+) cultivar showed a slightly higher number reads-per-million-reads (RPMR) of 22 nt in the mock-inoculated than in the TSWV-infected plants. In both treatments, the number of reads of 24 nt were higher than the other read lengths, but no difference between them. On the other hand, in the susceptible variety infected with TSWV, the number of reads of 21 and 22 nt were higher than in the mock-inoculated, but the number of reads of 23 and 24 nt was smaller. The 23 nt class showed a subtle difference in the susceptible variety, whereas the TSWV-infected plants had 50,000 RPMR less than the mock-inoculated (Figure 2). Similar results were obtained by Mitter et al. [29] and Margaria et al. [28] where they reported a higher number of siRNAs of 24 nt, followed by 21, 22, and 23 of endogenous siRNA, but a higher abundance of 21 and 22 nt vsiRNAs.

### 3.3. Hotspots in TSWV RNAs

**Figure 3:**
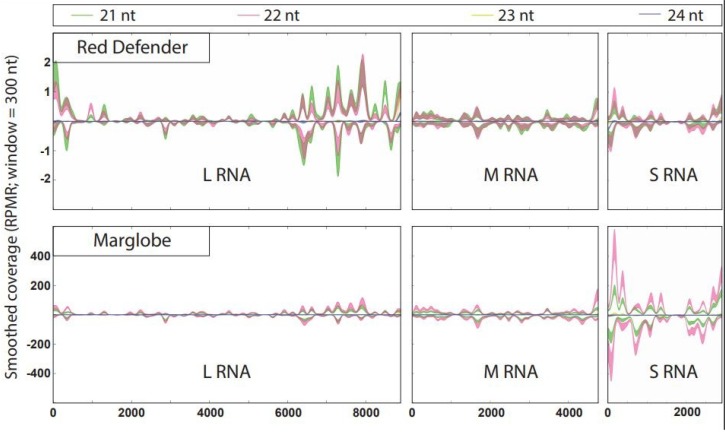
vsiRNAs profiles showing hotspots and corresponding regions in the tomato spotted wilt virus (TSWV) genomic RNAs in *Sw*-5(+) and *Sw*-5(−) varieties. The smoothed plots are standard error for each nucleotide size/alignment (i.e., the wider the red or green line, the greater the standard error of the 3 biological replicates at that position). The reads-per-million-reads (RPMR) scale for Red Defender is ±2.5 and for Marglobe is ±400.

The vsiRNAs were mapped to TSWV genomic RNAs L, M, and S in the *Sw*-5(+) and *Sw*-5(−) tomatoes (Figure 3). In the single nucleotide resolution map, the 21 to 24 nt vsiRNAs were mapped back to the three TSWV RNAs either in + or – sense. vsiRNAs were distributed throughout each of the three RNAs in both strands. In the resistant variety, the number of vsiRNAs were much higher from the L RNA, compared to M and S RNAs, whereas in case of susceptible genotype, the S RNA had a preponderance of vsiRNAs compared to those from L and M RNAs. The profiles of hotspots were quite similar for all the three RNAs in both genotypes with a trend to cover the 5′ and 3′ ends, and less coverage in the intergenic region (IGR) of the M and S RNAs. Interestingly, the L RNA had a larger hotspot region at the 3′ on the genomic RNA covering more than 2000 nucleotides (Figure 3). In the *Sw*-5(+) tomato, the hotspots in the L RNA had more abundance of vsiRNAs of 21 nt contrasting with the same spots in the *Sw*-5(−). The hotspot graph showed a higher number vsiRNAs aligned with the L RNA of *Sw*-5(+) cultivar, in comparison with the other RNAs, while in *Sw*-5(−) cultivar, the S RNA was targeted more in comparison to the other RNAs. However, the hotspot enrichment pattern was similar for the M RNA in both varieties. (Figure 3).

**Figure 4:**
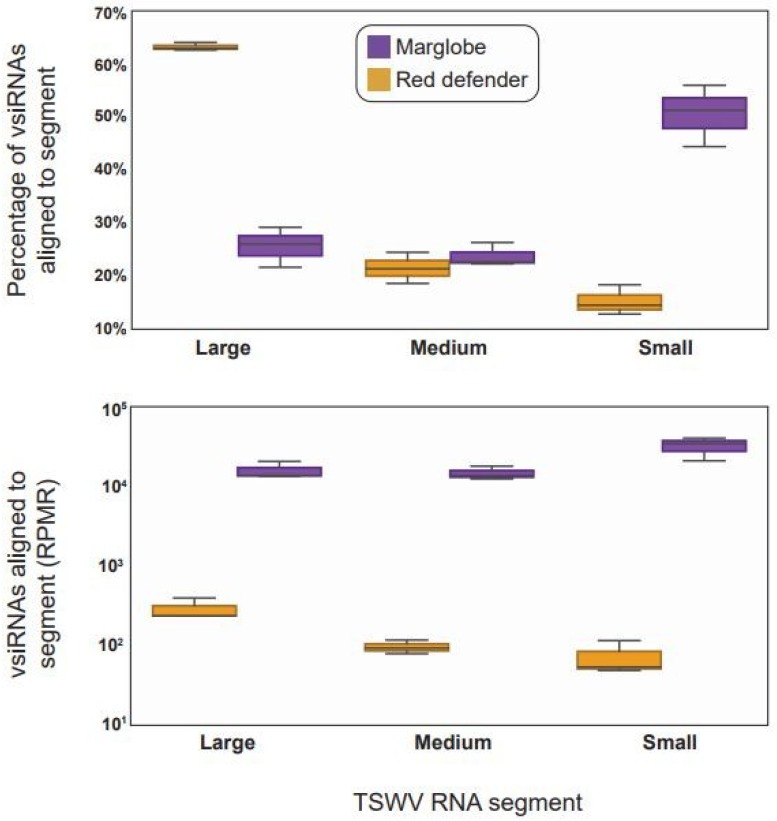
Boxplots of the viral-derived small interfering RNAs matching the segments of tomato spotted wilt virus (TSWV). Upper diagram corresponds to the percentage of vsiRNAs matching the Large, Medium, and Small RNAs. Bottom diagram corresponds to the reads-per-million-reads (RPMR) matching the three TSWV RNAs. Each plot corresponds to the average of three biological replicates with standard error bars. Purple corresponds to tomato cv. Marglobe, the susceptible genotype *Sw*-5(−), while the yellow line corresponds to cv. Red Defender, the resistant genotype, *Sw*-5(+). ANOVA analysis followed by Tuckey Honest Significant Difference (HSD) test (*p*-value < 0.05).

The boxplots (Figure 4) showed the highest percentage of vsiRNAs aligned to the L RNA in *Sw*-5(+) cultivar in comparison with the *Sw*-5(−) cultivar, but also when compared with the other RNAs. On the contrary, the percentage of the vsiRNAs aligned with the S RNA were higher in *Sw*-5(−) compared to the resistant variety, and the other RNAs. Interestingly, the M RNA was processed in similar proportions in both varieties. This result suggested a tendency to target the RNAs differentially in a genotype (resistant versus susceptible)-dependent manner. However, it is important to note that as expected, the vsiRNAs processing was higher in the susceptible tomato than in the resistant one (Figure 4).

**Figure 5:**
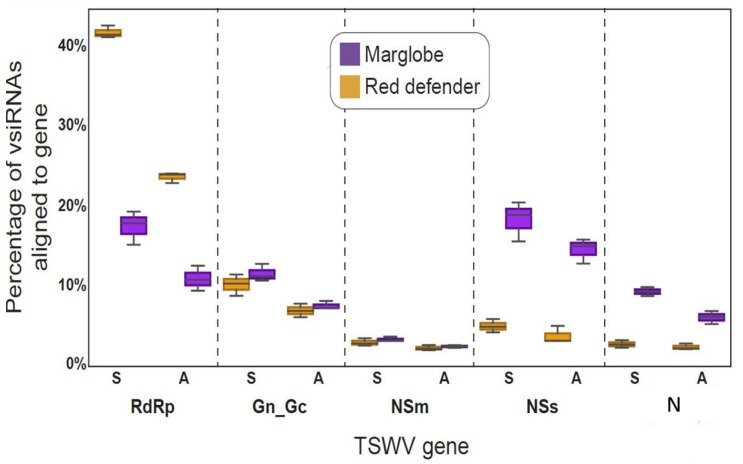
Boxplots of the percentage of viral-derived small interfering RNAs (vsiRNAs) aligned to each tomato spotted wilt virus (TSWV) gene in the tomato cv. Red Defender, *Sw*-5(+) and *Sw*-5(−) (cv. Marglobe). The smoothed plots are standard error for each range. RdRp: Polymerase gene; G_N_/G_C_: Glycoprotein precursor gene; NSm, Movement gene; NSs: silencing suppressor gene; N: Nucleoprotein gene. S: sense, A: antisense. Purple corresponds to tomato cv. Marglobe, the susceptible genotype *Sw*-5(−), while the yellow line corresponds to cv. Red Defender, the resistant genotype, *Sw*-5(+). ANOVA analysis followed by Tuckey HSD test (*p*-value <0.05).

The analysis of the alignment profiles of the vsiRNAs to each gene individually, shows that the percentage of reads aligned with the RdRp was higher in the resistant variety than in the susceptible one, and the percentage of reads for this gene, was significantly higher in the ORF sense than in the complementary sense in both genotypes. This trend agrees with the previous analysis by genomic RNA coverage. In case of G_N_/G_C_ and the NSm genes located on the M RNA, the percentage of vsiRNAs aligned was quite similar in both varieties, but with a higher percentage in the glycoprotein precursor gene than in the movement protein gene. However, in G_N_/G_C,_ the percentage of vsiRNAs was higher in the sense than in the complementary sense, but for NSm there was not difference in the coverage in both orientations. Regarding the genes located on the S RNA, the NSs and the N, the vsiRNAs processing was higher in the susceptible tomato than in the resistant, as was for the S RNA analysis. In the susceptible variety, the percentage of vsiRNAs was very high for the NSs, even higher than for the N; however, in both cases the sense strand had a higher percentage than the complementary sense. On the other hand, in the resistant variety, the processing of NSs and N did not show significant difference, and was same in the sense and antisense coverage (Figure 5). This result again shows a genotype-dependent preference for processing the genes of TSWV, where in the resistant variety, the polymerase gene is highly targeted, while in the susceptible variety, the NSs followed by the N gene are preferentially processed.

### 3.4. siRNAs 5′ Terminal Nucleotide-Strand Polarity

**Figure 6:**
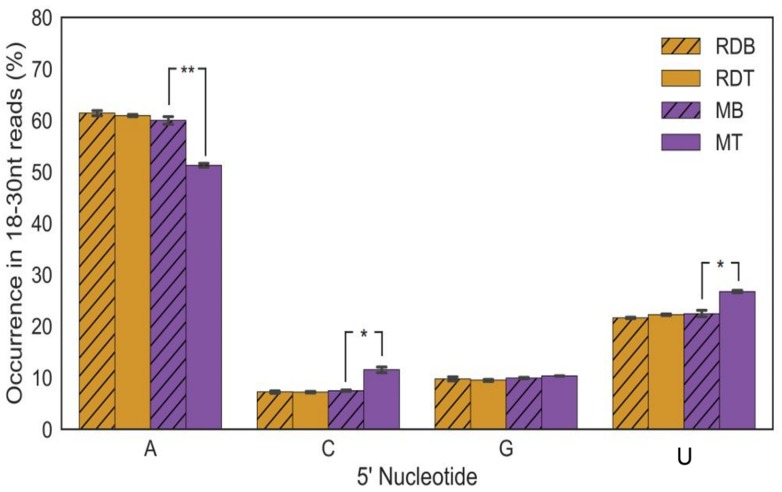
Percentage of tomato spotted wilt virus (TSWV) siRNA 5′ nucleotide enrichment. Purple corresponds to tomato Marglobe, the susceptible genotype Sw-5(−), while the yellow line corresponds to Red Defender, the resistant genotype, Sw-5(+). There is no difference in 5′ nucleotide enrichment among vsiRNAs, but there are significant changes in small RNAs between mock- and TSWV-inoculated Marglobe. RDB: Buffer-inoculated Red Defender (Mock); RDT: TSWV-infected Red Defender; MB: Buffer-inoculated Marglobe (Mock); MT: TSWV-infected Marglobe. Pairwise t-tests with Bonferroni *p*-value correction, * *p* < 0.05; ** *p* < 0.005. Tests performed using custom Python scripts.

The nature of the 5′ nucleotide in the siRNAs varied significantly in the susceptible genotype. In the susceptible variety [*Sw*-5(−)], the reads with a 5′-adenine were more abundant in the mock-inoculated control compared to the TSWV-inoculated, whereas cytosine (C) and uracil (U) were more abundant in the TSWV-inoculated plants. No such difference was seen in case of virus-infected and mock-inoculated plants containing the *Sw*-5 gene. No difference was seen between TSWV-infected resistant and mock-inoculated control as well with the mock-inoculated control of the susceptible genotype (Figure 6). The siRNA with a 5′ end of G did not change in the presence of TSWV, or in the two different host genotypes. In short, TSWV infection in the susceptible variety induced a greater number of siRNAs with a 5′end of C and U, and a reduction of the A, while these changes did not occur in the resistant variety (Figure 6).

### 3.5. RT-qPCR of TSWV

As the primers designed can amplify either the genes from the mRNAs or the genomic RNAs, it was difficult to determine precisely whether the amplicon obtained was for the genomic RNA or from the mRNA. Similar point was raised by Rotenberg et al. [38]) and Badillo-Vargas et al. [39] with respect to the TSWV-N gene or RNA primers. We agree with this point of view and choose to refer as virus and the gene acronym to the respective amplicon. Amplification of TSWV genes in inoculated tomato leaves showed a higher level of viral gene expression in the susceptible variety than in the resistant cultivar.

**Figure 7:**
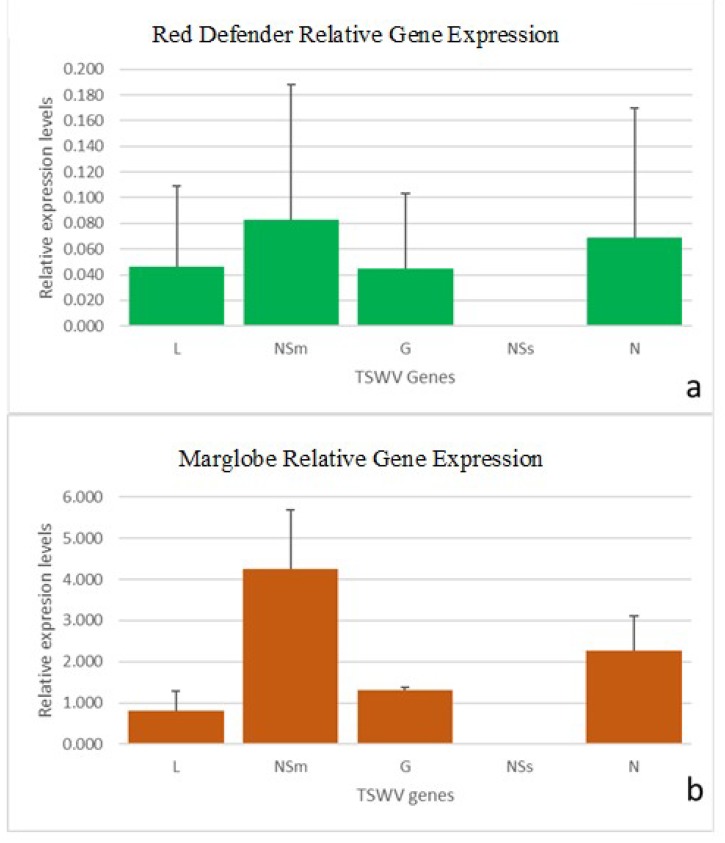
Relative expression of tomato spotted wilt virus (TSWV) genes in two tomato varieties, (**a**) The resistant variety Red Defender inoculated with TSWV. (**b**) The susceptible variety Marglobe inoculated with TSWV. Elongation factor 1-alpha (EF1a) and Glyceraldehyde 3-phosphate dehydrogenase GAPDH were used as the host genes for normalization. Error bar indicates the standard deviation. The bar correspondes to a delta-Ct nomalized Cts of the three biological replicates and three technical replicates per each one. L: Polymerase; NSm: Movement protein; G: Glycoprotein precursor; N: Nucleoprotein; NSs: Silencing suppressor.

EF1a and GAPDH were chosen as the housekeeping genes to normalize the gene expression as they showed stable expression among the experimental treatments. The primers designed for L, NSm, G2 and NSs genes (this study), and the primer pair for N gene from Rotenberg et al. [38] worked optimally, showing a single peak melting curve (Supplementary figure S1). All five genes exhibited differences of expression levels in *Sw*-5(−) and in *Sw*-5(+) cultivar, with NSm showing most dramatic difference (Figure 7). The relative expression level of NSs was very low in comparison with the other genes both in *Sw*-5(−) and *Sw*-5(+). Comparative analysis of all genes showed a highest level of the NSm gene followed by N, and with lowest expression levels of L and NSs in *Sw*-5(−). A similar pattern was found in *Sw*-5(+), but with a very low expression of all genes compare to *Sw*-5(−) genotype (Figure 7). The normal distribution of the gene expression data was analyzed by a Shapiro–Wilk test, where the null hypothesis of normal distribution was accepted for N, NSm, and NSs, but reject for G (supplementary Table S2). One-way ANOVA was conducted after the normalization validation, and only the N gene showed significantly differences between both genotypes. Additionally, when compare all the genes in the susceptible variety, the TSWV-N level was significantly higher in comparison with the other genes.

## 4. Discussion

Until now, the vsiRNA profiles of tospoviruses were studied in susceptible hosts and in systemically infected, non-inoculated leaves [28,30,32,33,34]. Here we profiled the comparative vsiRNAs populations in inoculated leaves of a resistant and a susceptible cultivar. TSWV and other tospoviral vsiRNAs profiles were determine in *N. benthamiana*, TSWV susceptible tomato, and peanut [28,29,30,32]. TSWV induced differential accumulation of vsiRNAs in similar combination of hosts, but in the case of tomato in a different variety [28,29]. Mitter et al. [29] in tomato and *N. benthamiana*, and Fletcher et al. [30] in peanut found 21 nt as the major class of vsiRNAs followed by the 22 nt, and the 24 nt. However, another tospovirus infecting tomato and tobacco, *Polygonum ringspot tospovirus* (PolRSV) in tomato showed the highest vsiRNAs correspond to 22 nt followed by 21 nt. While the virus induced similar accumulation of sRNAs in both hosts [32], interestingly in our study, the resistant variety showed a slight difference in the 22 nt, and no difference in the 21 nt, but the susceptible variety showed a perceptible difference in both sizes for mock- and TSWV-inoculated plants. In systemically infected, non-inoculated symptomatic leaves of *N. benthamiana* and susceptible tomato (cv. Sunny), at 17 dpi, Mitter et al. [29] found the profile of vsiRNAs in terms of relative abundance 21, 22, and 24 nt class size was similar in both the hosts. Interestingly, the number of vsiRNAs reads detected was higher in tomato than in *N. benthamiana.* Noticeably, TSWV induced a reduction in the peak of 24 nt in peanut, but a slight increase in the peak of 22 nt [30]. Similarly, our results showed that a reduction in the 24 nt peak in the susceptible cultivar, while the resistant cultivar did not show this reduction and in fact the pattern was the same as that of the mock control.

The size of the siRNAs is related to the type of DCL that is acting, for example DCL4 produces siRNAs of 21 nt, while the 22 nt are produced by DCL2 [42]. In our study, the abundance of 22 nt over the 21 suggest the predominant activity of DCL2 over DCL4. Studies in other viruses and hosts have shown the accumulation of predominantly 22 nt [43,44,45], while potyviruses such as *Cassava brown streak virus* accumulated predominantly 21 nt class in cassava cultivars [46]. In TSWV, M, and S RNAs possess intergenic regions that form a hairpin structure, while this is absent in the L RNA. According to Fusaro et al. [47], a hairpin structure of *Cucumber mosaic virus* satellite RNA was targeted more efficiently by DCL4. In TSWV, the silencing suppressor binds to long and short dsRNA molecules and suppresses local and systemic silencing, but also acts in the biogenesis of siRNAs by sequestration [27]. The specific activity of the NSs (in PolRSV) could have a role in determining the differential vsiRNAs profiles [32].

On the other hand, DCL3 is involved in the production of 24 nt siRNAs, which indicates more activity in resistant tomato in both scenarios: mock- and TSWV-inoculated, and in the susceptible variety’s mock control. Interestingly, TSWV-infected Marglobe showed a lower peak compared to the 22 nt peak. This could be an indication that in compatible interactions, the activity of DCL3 can be reduced compared to a more prioritized activity of DCL2. Similar results were evident in susceptible host-virus interactions [28,32,48]. In the *Sw*-5(+) tomato, some of the hotspots in L RNA had more abundance of vsiRNA of 21 nt contrasting with the same spots in the *Sw*-5(−). Besides the low number of RPMR, this could indicate a more modest action of the DCL4. It could be the presence of another unknown mechanism that blocked the virus replication before the necrotic response reached a level that confines the virus to the infected cells thus preventing the virus spread to neighboring cells.

Fletcher et al. [30] found a higher number of vsiRNA hotspots in the L RNA segment corresponding to the RdRp gene in peanut while the susceptible tomato cv. Sunny and tobacco lack this vsiRNAs coverage. Margaria et al. [28] found that in the absence of a functional silencing suppressor, a hotspot happens in the L RNA located at 6100 to 8000 bp position, mainly in the antisense strand. However, the hotspot distribution was similar for the M and S RNAs, in both TSWV isolates suggesting a role of the NSs protecting the L RNA from degradation. Similar results were reported by Mitter et al. [29] with a very low processing of L RNA in tomato and tobacco. The pattern of the vsiRNAs matching the TSWV RNAs in tomato *Sw*-5(+) peanut was more similar to the one reported by Fletcher et al. [30]. Moreover, Fletcher et al. [30] suggested that the host-specific vsiRNAs processing of TSWV-RdRp may have significant downstream impact on pathogenicity.

The *Sw*-5 gene is known to reduce/control the movement of the virus. It was surprising that the percentage of vsiRNAs aligned with the NSm was quite similar in both varieties; however, the number of RPMR was higher in the susceptible variety. On the other hand, in the resistant variety, the percentage of vsiRNAs processed, the number of reads was similar between NSs and the N. Interestingly, the polymerase gene had the highest percentage of vsiRNAs in *Sw*-5(+) in the viral sense. This led us to hypothesize a complementary action in the plant immune machinery to degrade the corresponding RdRp supporting the function of the *Sw*-5 (=limiting the movement of the virus from cell to cell), or it could be due to a lack of TSWV protective mechanism for the polymerase, reducing its function in producing replicative viral forms. It would be like an early recognition of the NSm protein by the *Sw*-5 protein, the immune response consisted of the activation of the programmed cell death (PCD) but also a silencing interference machinery that quickly targets the polymerase transcripts, resulting in the high percentage of vsiRNAs aligned with the RdRp in sense polarity.

The higher number to vsiRNAs matching the NSs and the N in the susceptible host can be an indication of either a highly efficient amplification phase focused on those RNAs, or a potential use of the resulting vsiRNAs targeting host genes to allow the viral life infection cycle. This topic is gaining scientific interest, as it has been discovered as a secondary route that the virus used to counterattack the antiviral plant defense by hijacking the host genes.

Interestingly, Bai et al. [49], in TYLCV-susceptible tomato, found that the frequency of vsiRNAs directly correlated with the viral transcript level of the corresponding RNAs. The relative levels of the silencing suppressor gene found by RT-qPCR did not correspond to the predicted levels of the corresponding vsiRNAs. While in the susceptible genotype, the vsiRNAs aligned to the NSs were higher than those for N; however, the relative levels accumulation of N was higher than that of the NSs. The relative levels S RNA was higher than that of M RNA, which agreed with the prediction of the vsiRNAs population. The lower levels of accumulation of NSs detected by RT-qPCR could be the result of a lower efficiency of the primers used, or could reflect the actual level of the corresponding RNA at that specific moment of infection. Interestingly, the above trend in the frequency of the TSWV genes was similar between the susceptible and resistant varieties.

The fewer number of the vsiRNAs in the resistant variety can also be due to a mechanism that quickly counters the NSs thus limiting the production of RdRp, and resulting in fewer dsRNA replicative forms before the HR is triggered. It would be interesting to determine the siRNA biogenesis close to the time of infection (3 to 6 h post-inoculation), and subsequently a time series such as one, three, five days, etc. Similar results were found in resistant genotypes of cassava infected with Ugandan cassava brown streak virus [46], and in tomato infected with TYLCV [49] where resistant genotypes produced fewer amounts of vsiRNAs in comparison with the susceptible ones. Moreover, Spanò et al. [50] determined the resistance response to TSWV RB isolate on a suscepitble tomato cultivar grafted onto a resistant roostock. The observed resistance was potentially the result of a RNAi respose that migrated from the rootstock to scion, triggering the expression of RNA-silencing genes.

It is assumed that the main role of vsiRNAs is to target viral mRNA molecules to inhibit viral replication [25]. The RNA-silencing signal is mobile, but the mechanism remains unclear. It is likely that it would follow the phloem transport or the cell to cell movement as in the experiments using grafted plants—from a silenced rootstock to a non-silenced scion, or from a resistant genotype to a susceptible one, and vice-versa [50,51].

Fletcher et al. [30] pointed that the disruption of sRNA levels and the potential for NSs action on plant-generated sRNAs by perturbation of target RNA transcript induced phenotypical changes in the host. In this study, the abundance of NSs vsiRNAs in the susceptible tomato, in comparison with the resistant one, combined with the low levels of the NSs RNA detected, could be an indication of the role of the NSs-derived vsiRNAs in the interference with the host machinery to trigger the optimal cellular conditions for TSWV proliferation. On the other hand, the abundance of the vsiRNAs matching the TSWV-RdRp in the resistant genotype could be an indication of a hierarchical targeting of the host machinery against this specific target as a complement of the effector-trigger immunity (ETI) HR that needs to be investigated.

## Figures and Tables

**Table 1:** Number of reads predicted from tomato varieties with or without *Sw*-5 following inoculation by TSWV or buffer (mock). Each value is the average of three replicates, after clean reads from 18 to 34 nucleotides.

	*Sw*-5(+) Mock	*Sw*-5(+) TSWV	*Sw*-5(−) Mock	*Sw*-5(−) TSWV
Total sequences *	30,796,029.33	29,959,930.67	29,738,796.33	29,284,469.33
Total Nucleotides in data set *	701,391,986.7	682,215,963.3	674,277,615.3	652,956,838.3

* Processed with CLC genomics.

## Supplementary Materials

The following are available online at https://www.mdpi.com/1999-4915/12/4/363/s1, Supplementary Table S1: Primers used to amplify tomato spotted wilt virus and tomato host reference genes by RT-qPCR. Supplementary Table S2: Relative absorbance values (average of two wells) at 405 nm from DAS-ELISA of TSWV in younger non-inoculated leaves of resistant cultivar, Red Defender. Supplementary Table S3: Shapiro-Wilk test output for relative expression of TSWV genes. Supplementary Figure S1. Effect on growth development of TSWV infection in resistant Red Defender and susceptible Marglobe varieties. Supplementary Figure S2. Melting curves of tomato spotted wilt virus (TSWV) genes.
