# Supplementary material for: The Tomato spotted wilt virus (TSWV) Genome is Differentially Targeted in TSWV-Infected Tomato (Solanum lycopersicum) with or without Sw-5 Gene
Source: Viruses. 2020 Mar 26;12(4):363. doi: 10.3390/v12040363 (PMC7232525; doi:10.3390/v12040363)
Supplement: Supplementary file 1 [file viruses-12-00363-s001.pdf]

Supplementary Table 1. Primers used to amplify tomato spotted wilt virus and tomato host reference genes by RT-qPCR.

Gene	Name	Direction	Sequence 5′-3′	PCR Product (bp)	Reference
Polymerase	qTSWV-L-4F	Forward	GTTGCACGGAATCTTCGCAT	123	This Study
	qTSWV-L-4R	Reverse	TGCACAGCCCCATAAGCAAA		
Glycoprotein	qTSWV-G-2F	Forward	AATGTTCTAACTGTGGCAATTTGTG	135	This Study
	qTSWV-G-2R	Reverse	CGTAGCCGTGATCTGTTTCTTTG		
Movement Protein	qTSWV-NSm-F	Forward	TCCCAACATGCCATCTGAAAAGC	168	This Study
	qTSWV-NSm-R	Reverse	ACCCCCTTCTTGTATTCTTGGCTG		
Nucleocapsid gene	TSWV-N-Rot-F	Forward	GCTTCCCACCCTTTGATTC	139	[38]
	TSWV-N-Rot-R	Reverse	ATAGCCAAGACAACACTGATC		
Silencing Suppressor	qTSWV-NSs-F	Forward	TCCTGCTCAGCTCCACTCTATTG	138	This Study
	qTSWV-NSs-R	Reverse	TTGACACAAGACAAAAAGCCTCTG		
Ubiquitin 3	UBI-F	Forward	TCGTAAGGAGTGCCCTAATGCTGA	119	[40]
	UBI-R	Reverse	CAATCGCCTCCAGCCTTGTTGTAA		
Glyceraldehyde 3-phosphate dehydrogenase	GAPDH-F	Forward	ACCACAAATTGCCTTGCTCCCTTG	110	[40]
	GAPDH-R	Reverse	ATCAACGGTCTTCTGAGTGGCTGT		
Uridylate Kinase	UBI-F	Forward	TGGTAAGGGCACCCAATGTGCTAA	107	[40]
	UBI-R	Reverse	ATCATCGTCCCATTCTCGGAACCA		
Elongation factor -1 alpha	EF-1a-F	Forward	GATTGGTGGTATTGGAACTGTC	129	[41]
	EF-1a-R	Reverse	AGCTTCGTGGTGCATCTC		

Supplementary Table 2. Relative absorbance values (average of two wells) at 405 nm from DAS-ELISA of TSWV in younger non-inoculated leaves of resistant cultivar, Red Defender.

**Treatment**	**Absorbance at 405 nm at 60 minutes**
Buffer control	0.094
Negative Control	0.098
TSWV Positive control	1.084
Red Defender/Buffer/#1/systemic	0.094
Red Defender/Buffer/#2/systemic	0.094
Red Defender/Buffer/#3/systemic	0.093
Red Defender/Buffer/#4/systemic	0.098
Red Defender/Buffer/#6/systemic	0.092
Red Defender/Buffer/#7/systemic	0.094
Red Defender/Buffer/#8/systemic	0.097
Red Defender/Buffer/#5/systemic	0.095
Red Defender/Buffer/#9/systemic	0.095
Red Defender/TSWV/#1/systemic	0.096
Red Defender/TSWV/#2/systemic	0.099
Red Defender/TSWV/#3/systemic	0.106
Red Defender/TSWV/#4/systemic	0.104
Red Defender/TSWV/#5/systemic	0.098
Red Defender/TSWV/#6/systemic	0.095
Red Defender/TSWV/#7/systemic	0.098
Red Defender/TSWV/#8/systemic	0.094
Red Defender/TSWV/#9/systemic	0.095
Red Defender/TSWV/#10/systemic	0.099
Red Defender/TSWV/#11/systemic	0.097
Red Defender/TSWV/#12/systemic	0.093
Red Defender/TSWV/#13/systemic	0.088
Red Defender/TSWV/#14/systemic	0.098
Red Defender/TSWV/#15/systemic	0.099
Red Defender/TSWV/#16/systemic	0.101
Red Defender/TSWV/#17/systemic	0.101
Red Defender/TSWV/#18/systemic	0.102
Red Defender/TSWV/#19/systemic	0.095
Red Defender/TSWV/#20/systemic	0.096
Red Defender/TSWV/#21/systemic	0.086
Red Defender/TSWV/#22/systemic	0.105
Red Defender/TSWV/#23/systemic	0.123
Red Defender/TSWV/#24/systemic	0.125
Red Defender/TSWV/#25/systemic	0.127
Red Defender/TSWV/#26/systemic	0.119
Red Defender/TSWV/#27/systemic	0.120

Supplementary Table 3. Shapiro-Wilk test output for relative expression of TSWV genes. The Prob < W value listed in the output is the p-value at alpha of 0.05.

**Gene**	**W**	**Prob<W**
G	0.790131	0.0478
NSm	0.798916	0.0575
N	0.832862	0.1136
NSs	0.884421	0.3298

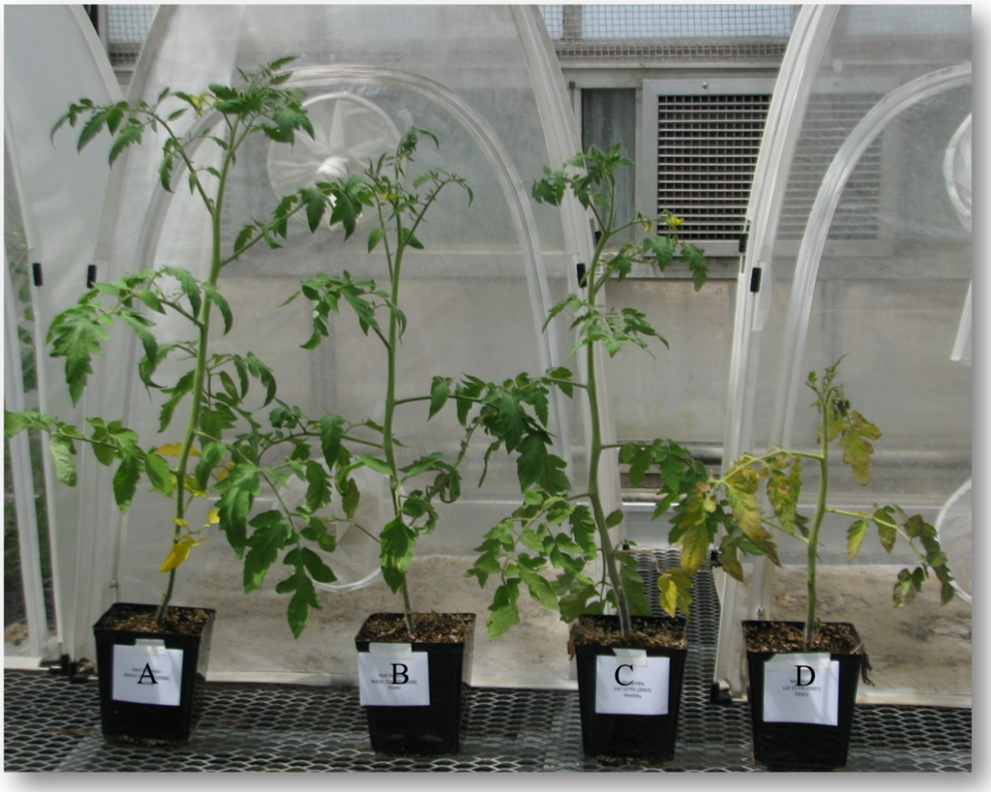

Supplementary Figure 1. Effect on growth development of TSWV infection in resistant Red Defender and susceptible Marglobe varieties. A) Mock inoculated resistant genotype; B) TSWV inoculated resistant genotyper; C) Mock inoculated susceptible genotype: D) TSWV inoculated suscetiple genotype.

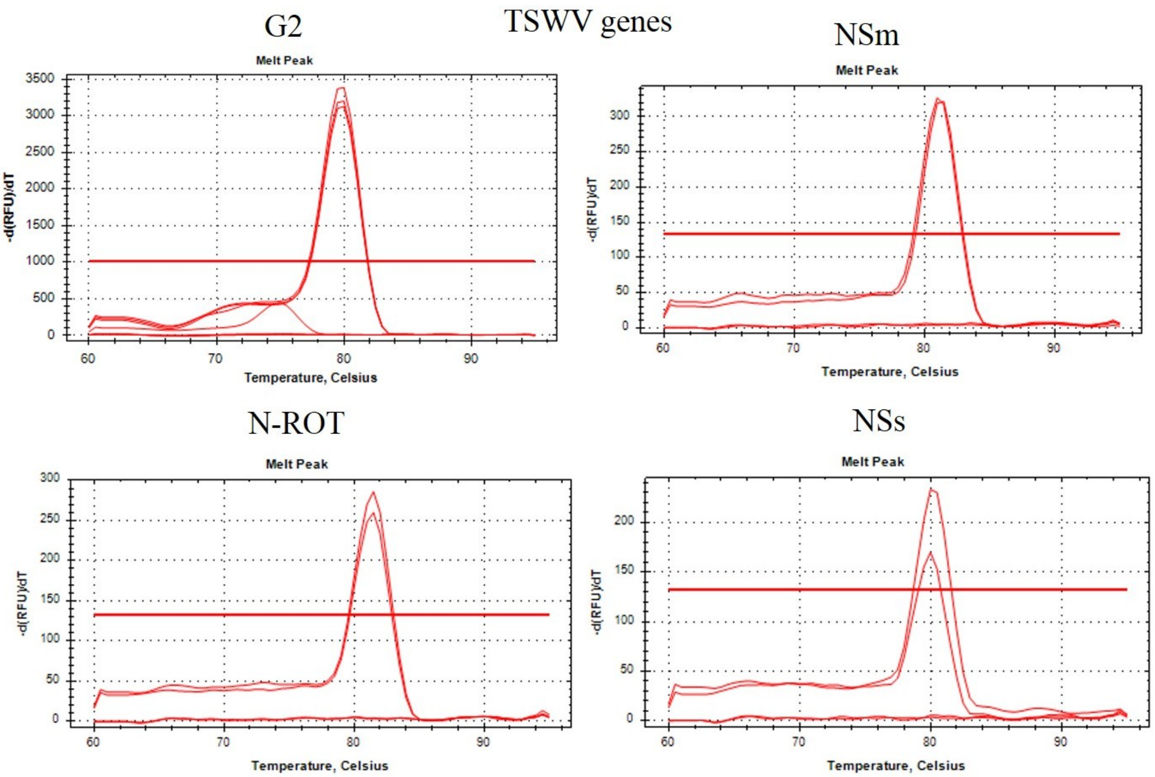

Supplementary Figure 2 . Melting curves of tomato spotted wilt virus (TSWV) genes. G2: GN/GC precursor; NSm: cell to cell movement; N-ROT: for the nucleoprotein; NSs: silencing suppressor.
